# Supplementary material for: Evaluation of the facial profile of skeletal Class III patients undergoing camouflage orthodontic treatment: a retrospective study
Source: PeerJ. 2024 Jul 24;12:e17733. doi: 10.7717/peerj.17733 (PMC11283169; doi:10.7717/peerj.17733)
Supplement: Supplemental Information 2 [file peerj-12-17733-s002.docx]

Supplemental Table 1 The Definitions of the Cephalometric Measurements.

|  | Measurements | Scale | Definitions |
| --- | --- | --- | --- |
| Soft tissue measurements | Z Angle | deg | Angle between Frankfort plane and a line through the pogonion and most prominent point of upper or lower lip |
|  | Lower Lip-E line | mm | Distance from Labrale inferius (Li) to E-line |
|  | Upper Lip-E Line | mm | Distance from Labrale superius (Ls) to E-line |
|  | Nose Prominence | mm | Distance between the tip of the nose and a perpendicular line drawn to the Frankfort plane from the vermilion |
|  | Nasolabial Angle | deg | Angulation between the lower border of nose and upper lip tangent |
|  | MentoLabial Angle | deg | Angulation between the soft tissue chin and lower lip tangent |
| Skeletal tissue measurements | ANB | deg | Angulation between NA and NB |
|  | SNA | deg | Angulation between NA and sella-nasion (SN) plane |
|  | SNB | deg | Angulation between NB and sella-nasion (SN) plane |
|  | MP/SN | deg | Angulation between SN plane and the mandibular plane |
|  | MP/FH | deg | Angulation between Frankfort plane (FH) and the mandibular plane |
|  | Gonial Jaw Angle | deg | Angulation between S-Ar and Ar-Go |
|  | Y Axis | deg | Y-axis (S-Gn) angulation to SN plane |
|  | LFH | % | Lower face height divided by Total face height (ANS-Me:N-Me) |
|  | Pog-NB | mm | Distance from Pg to NB |
|  | OP/SN | deg | Angulation between Occlusal Plane to SN plane |
|  | Wits | mm | Distance between AO and BO (foot of perpendicular line from A/B point to OP plane) |
| Dental measurements | U1/SN | deg | Upper incisor angulation to SN plane |
|  | U1/AP | deg | Upper incisor angulation to A-Pg plane |
|  | U1-AP | mm | Distance from maxillary incisor tip to A-Pg plane |
|  | U1/NA | deg | Upper incisor angulation to NA |
|  | U1-NA | mm | Distance from maxillary incisor tip to NA |
|  | L1/MP | deg | Lower incisor angulation to mandibular plane |
|  | L1/AP | deg | Lower incisor angulation to A-Pg plane |
|  | L1-AP | mm | Distance from lower incisor tip to A-Pg plane |
|  | L1/NB | deg | Lower incisor angulation to NB |
|  | L1-NB | mm | Distance from lower incisor tip to NB |
|  | U1/L1 | deg | Interincisal Angle |
|  | FA-Fall | mm | Distance from FA point of upper incisor to FALL |
|  | overjet | mm | Horizontal distance from maxillary incisor tip to the labial surface of the lower incisor |
