# Supplementary material for: Evaluation of the facial profile of skeletal Class III patients undergoing camouflage orthodontic treatment: a retrospective study
Source: PeerJ. 2024 Jul 24;12:e17733. doi: 10.7717/peerj.17733 (PMC11283169; doi:10.7717/peerj.17733)
Supplement: Supplemental Information 3 [file peerj-12-17733-s003.docx]

| **Supplemental Table 2. Analysis of normality distribution** | | | | | | |
| --- | --- | --- | --- | --- | --- | --- |
|  | Kolmogorov-Smirnov^a^ | | | Shapiro-Wilk | | |
|  | statistic | df | Sig. | statistic | df | Sig. |
| Z Angle | .053 | 80 | .200^*^ | .988 | 80 | .634 |
| Lower Lip-E line | .057 | 80 | .200^*^ | .985 | 80 | .453 |
| Upper Lip-E Line | .057 | 80 | .200^*^ | .993 | 80 | .930 |
| Nose Prominence | .051 | 80 | .200^*^ | .986 | 80 | .521 |
| Nasolabial Angle | .051 | 80 | .200^*^ | .991 | 80 | .840 |
| MentoLabial Angle | .049 | 80 | .200^*^ | .987 | 80 | .596 |
| ANB# | .188 | 80 | .000 | .877 | 80 | .000 |
| SNA | .076 | 80 | .200^*^ | .985 | 80 | .463 |
| SNB | .060 | 80 | .200^*^ | .974 | 80 | .108 |
| MP/SN | .075 | 80 | .200^*^ | .984 | 80 | .413 |
| MP/FH | .095 | 80 | .069 | .987 | 80 | .582 |
| Gonial Jaw Angle | .081 | 80 | .200^*^ | .978 | 80 | .196 |
| Y Axis | .077 | 80 | .200^*^ | .964 | 80 | .024 |
| LFH | .074 | 80 | .200^*^ | .968 | 80 | .043 |
| Pog-NB | .122 | 80 | .005 | .972 | 80 | .076 |
| OP/SN | .092 | 80 | .092 | .975 | 80 | .110 |
| Wits# | .199 | 80 | .000 | .833 | 80 | .000 |
| U1/SN | .063 | 80 | .200^*^ | .986 | 80 | .562 |
| U1/AP | .059 | 80 | .200^*^ | .990 | 80 | .786 |
| U1-AP | .071 | 80 | .200^*^ | .987 | 80 | .607 |
| U1/NA | .064 | 80 | .200^*^ | .988 | 80 | .696 |
| U1-NA | .084 | 80 | .200^*^ | .976 | 80 | .132 |
| L1/MP | .047 | 80 | .200^*^ | .977 | 80 | .149 |
| L1/AP | .075 | 80 | .200^*^ | .986 | 80 | .527 |
| L1-AP | .055 | 80 | .200^*^ | .984 | 80 | .420 |
| L1/NB | .069 | 80 | .200^*^ | .985 | 80 | .502 |
| L1-NB | .073 | 80 | .200^*^ | .985 | 80 | .479 |
| Interincisal Angle | .069 | 80 | .200^*^ | .988 | 80 | .634 |

^#^ ANB, and Wits were shown as a skewed distribution, the correlations between subjective VAS scores and objective measurements were assessed using Spearman correlation.
