# Supplementary material for: Evaluation of the facial profile of skeletal Class III patients undergoing camouflage orthodontic treatment: a retrospective study
Source: PeerJ. 2024 Jul 24;12:e17733. doi: 10.7717/peerj.17733 (PMC11283169; doi:10.7717/peerj.17733)
Supplement: Supplemental Information 4 [file peerj-12-17733-s004.docx]

Supplemental Table 3 Pearson Correlation Between Visual Analog Scale (VAS) Score of Pretreatment Profile and 30 Cephalometric Measurements in High Angle patients and Non-high Angle patients

| High Angle (n=29) | | | | Non-high angle (n=51) | | | |
| --- | --- | --- | --- | --- | --- | --- | --- |
| Variable | r | P | Order | Variable | r | P | Order |
| L1-AP | -0.559 | 0.002* | 1 | L1-AP | -0.385 | 0.005* | 1 |
| L1-NB | -0.555 | 0.002* | 2 | L1-NB | -0.361 | 0.009* | 2 |
| L1/AP | -0.522 | 0.004* | 3 | LowerLip-E line | -0.337 | 0.016* | 3 |
| L1/NB | -0.518 | 0.004* | 4 | Z Angle | 0.296 | 0.035* | 4 |
| L1/MP | -0.488 | 0.007* | 5 | L1/NB | -0.280 | 0.046* | 5 |
| LowerLip-E line | -0.479 | 0.009* | 6 | L1/AP | -0.278 | 0.048* | 6 |
| Interincisal Angle | 0.451 | 0.014* | 7 | L1/MP | -0.277 | 0.049* | 7 |
| UpperLip-E line | -0.449 | 0.014* | 8 | overjet | -0.275 | 0.051 | 8 |
| Nasolabial Angle | 0.407 | 0.028* | 9 | Nose Prominence | -0.235 | 0.097 | 9 |
| U1-AP | -0.309 | 0.103 | 10 | Interincisal Angle | 0.222 | 0.118 | 10 |
| FA-Fall | -0.290 | 0.128 | 11 | MP/SN | 0.173 | 0.226 | 11 |
| U1-NA | -0.288 | 0.130 | 12 | Y Axis | 0.146 | 0.308 | 12 |
| Z Angle | 0.236 | 0.219 | 13 | Gonial Jaw Angle | -0.144 | 0.313 | 13 |
| U1/AP | -0.235 | 0.220 | 14 | U1-AP | -0.136 | 0.342 | 14 |
| U1/SN | -0.233 | 0.224 | 15 | MentoLabial Angle | -0.124 | 0.386 | 15 |
| Nose Prominence | 0.232 | 0.225 | 15 | Nasolabial Angle | 0.120 | 0.401 | 16 |
| LFH | 0.231 | 0.227 | 17 | U1-NA | -0.118 | 0.409 | 17 |
| U1/NA | -0.219 | 0.253 | 18 | Pog-NB | 0.101 | 0.482 | 18 |
| Gonial Jaw Angle | 0.209 | 0.277 | 19 | MP/FH | -0.087 | 0.544 | 19 |
| SNA | -0.177 | 0.358 | 19 | FA-Fall | -0.085 | 0.552 | 20 |
| Pog-NB | 0.175 | 0.364 | 21 | SNA | -0.084 | 0.557 | 21 |
| OP/SN | 0.167 | 0.386 | 22 | Wits^#^ | -0.079 | 0.562 | 22 |
| SNB | -0.162 | 0.401 | 23 | U1/SN | -0.080 | 0.577 | 23 |
| Wits^#^ | -0.142 | 0.578 | 24 | SNB | -0.074 | 0.608 | 24 |
| MP/FH | -0.090 | 0.644 | 25 | U1/AP | -0.073 | 0.609 | 25 |
| MentoLabial Angle | 0.085 | 0.663 | 26 | LFH | 0.064 | 0.656 | 26 |
| overjet | 0.068 | 0.727 | 27 | U1/NA | -0.060 | 0.676 | 27 |
| Y Axis | 0.039 | 0.840 | 28 | ANB^#^ | -0.022 | 0.751 | 28 |
| ANB^#^ | -0.022 | 0.890 | 29 | UpperLip-E line | 0.016 | 0.910 | 29 |
| MP/SN | -0.005 | 0.980 | 30 | OP/SN | 0.010 | 0.942 | 30 |

^#^ ANB, and Wits were shown as a skewed distribution, the correlations between subjective VAS scores and objective measurements were assessed using Spearman correlation.
