# Supplementary material for: Evaluation of the facial profile of skeletal Class III patients undergoing camouflage orthodontic treatment: a retrospective study
Source: PeerJ. 2024 Jul 24;12:e17733. doi: 10.7717/peerj.17733 (PMC11283169; doi:10.7717/peerj.17733)
Supplement: Supplemental Information 5 [file peerj-12-17733-s005.docx]

Supplemental Table 4 Pearson Correlation Between Visual Analog Scale (VAS) Score of Pretreatment Profile and 30 Cephalometric Measurements in Adolescent patients and Adult patients

| Adolescent (n=24) | | | | Adult (n=56) | | | |
| --- | --- | --- | --- | --- | --- | --- | --- |
| Variable | r | P | Order | Variable | r | P | Order |
| L1/AP | -0.494 | 0.014* | 1 | Pog-NB | 0.303 | 0.023* | 1 |
| L1-AP | -0.463 | 0.023* | 2 | L1-AP | -0.275 | 0.040* | 2 |
| Nasolabial Angle | 0.367 | 0.078 | 3 | LowerLip-E line | -0.260 | 0.053 | 3 |
| OP/SN | 0.348 | 0.095 | 4 | Gonial Jaw Angle | 0.240 | 0.074 | 4 |
| L1-NB | -0.339 | 0.105 | 5 | SNB | -0.240 | 0.075 | 5 |
| ANB# | 0.338 | 0.106 | 6 | SNA | -0.192 | 0.156 | 6 |
| LowerLip-E line | -0.291 | 0.167 | 7 | Nasolabial Angle | 0.185 | 0.173 | 7 |
| FA-Fall | 0.268 | 0.206 | 8 | ANB# | 0.183 | 0.177 | 8 |
| L1/NB | -0.257 | 0.226 | 9 | MP/FH | 0.152 | 0.262 | 9 |
| MentoLabial Angle | -0.231 | 0.277 | 10 | MP/SN | 0.152 | 0.263 | 10 |
| SNB | -0.224 | 0.293 | 11 | L1-NB | -0.151 | 0.267 | 11 |
| Interincisal Angle | 0.213 | 0.317 | 12 | UpperLip-E line | -0.132 | 0.333 | 12 |
| U1/NA | -0.198 | 0.354 | 13 | Y Axis | 0.121 | 0.374 | 13 |
| L1/MP | -0.196 | 0.360 | 14 | L1/NB | -0.115 | 0.398 | 14 |
| U1/SN | -0.191 | 0.372 | 15 | Z Angle | 0.112 | 0.410 | 15 |
| U1-NA | -0.183 | 0.392 | 16 | L1/MP | -0.105 | 0.440 | 16 |
| UpperLip-E line | 0.181 | 0.399 | 17 | Nose Prominence | -0.097 | 0.475 | 17 |
| overjet | -0.139 | 0.516 | 18 | L1/AP | -0.085 | 0.533 | 18 |
| LFH | -0.122 | 0.569 | 19 | U1-NA | 0.075 | 0.582 | 19 |
| Z Angle | 0.115 | 0.591 | 20 | LFH | 0.074 | 0.590 | 20 |
| MP/SN | 0.104 | 0.627 | 21 | OP/SN | -0.066 | 0.627 | 21 |
| Gonial Jaw Angle | 0.069 | 0.750 | 22 | U1/NA | 0.065 | 0.632 | 22 |
| U1/AP | 0.059 | 0.785 | 23 | U1-AP | 0.062 | 0.651 | 23 |
| Pog-NB | -0.054 | 0.801 | 24 | U1/AP | 0.050 | 0.714 | 24 |
| Y Axis | 0.048 | 0.823 | 25 | Wits# | -0.039 | 0.775 | 25 |
| MP/FH | -0.048 | 0.825 | 26 | MentoLabial Angle | 0.028 | 0.836 | 26 |
| Nose Prominence | -0.044 | 0.838 | 27 | FA-Fall | 0.026 | 0.849 | 27 |
| U1-AP | 0.044 | 0.839 | 28 | U1/SN | -0.025 | 0.857 | 28 |
| SNA | -0.038 | 0.859 | 29 | overjet | -0.011 | 0.933 | 29 |
| Wits# | 0.035 | 0.871 | 30 | Interincisal Angle | 0.009 | 0.949 | 30 |

^#^ ANB, and Wits were shown as a skewed distribution, the correlations between subjective VAS scores and objective measurements were assessed using Spearman correlation.
