# Supplementary material for: Evaluation of the facial profile of skeletal Class III patients undergoing camouflage orthodontic treatment: a retrospective study
Source: PeerJ. 2024 Jul 24;12:e17733. doi: 10.7717/peerj.17733 (PMC11283169; doi:10.7717/peerj.17733)
Supplement: Supplemental Information 6 [file peerj-12-17733-s006.docx]

Supplemental Table 5 Pearson Correlation Between Changes in Visual Analog Scale (VAS) Score and Changes in 30 Cephalometric Measurements of Adolescent patients and Adult patients

| Adolescent (n=24) | | | | Adult (n=56) | | | |
| --- | --- | --- | --- | --- | --- | --- | --- |
| Variable | r | P | Order | Variable | r | P | Order |
| ΔL1-AP | -0.436 | 0.033* | 1 | ΔL1-AP | -0.427 | 0.001** | 1 |
| ΔL1-NB | -0.423 | 0.039* | 2 | ΔLowerLip-E line | -0.401 | 0.002** | 2 |
| ΔL1/NB | -0.421 | 0.041* | 3 | ΔL1-NB | -0.390 | 0.003** | 3 |
| ΔL1/AP | -0.391 | 0.059 | 4 | ΔLFH | -0.350 | 0.008** | 4 |
| ΔL1/MP | -0.383 | 0.065 | 5 | ΔL1/AP | -0.318 | 0.017* | 5 |
| ΔMentoLabial Angle | -0.380 | 0.067 | 6 | ΔL1/MP | -0.308 | 0.021* | 6 |
| ΔLowerLip-E line | -0.342 | 0.102 | 7 | ΔL1/NB | -0.304 | 0.023* | 7 |
| ΔMP/FH | -0.337 | 0.108 | 8 | ΔInterincisal Angle | 0.304 | 0.023* | 8 |
| ΔWits^#^ | -0.300 | 0.155 | 9 | ΔZ Angle | 0.297 | 0.026* | 9 |
| ΔZ Angle | 0.275 | 0.194 | 10 | ΔMP/SN | -0.280 | 0.037* | 10 |
| ΔInterincisal Angle | 0.272 | 0.199 | 11 | ΔNasolabial Angle | 0.252 | 0.061 | 11 |
| ΔPog-NB | 0.264 | 0.212 | 12 | ΔU1/SN | -0.189 | 0.164 | 12 |
| Δoverjet | -0.237 | 0.265 | 13 | ΔU1-NA | -0.188 | 0.165 | 13 |
| ΔU1-AP | -0.224 | 0.293 | 14 | ΔFA-Fall | -0.177 | 0.191 | 14 |
| ΔMP/SN | -0.179 | 0.402 | 15 | ΔANB^#^ | 0.164 | 0.227 | 15 |
| ΔSNA | -0.170 | 0.427 | 16 | ΔU1/AP | -0.164 | 0.227 | 16 |
| ΔFA-Fall | -0.168 | 0.432 | 17 | ΔU1-NA | -0.159 | 0.242 | 17 |
| ΔANB^#^ | -0.164 | 0.445 | 18 | ΔU1-AP | -0.156 | 0.252 | 18 |
| ΔU1-NA | -0.155 | 0.470 | 19 | ΔY Axis | 0.153 | 0.260 | 19 |
| ΔLFH | -0.155 | 0.471 | 20 | ΔUpperLip-E line | -0.139 | 0.306 | 20 |
| ΔNasolabial Angle | 0.153 | 0.476 | 21 | ΔMentoLabial Angle | 0.138 | 0.310 | 21 |
| ΔU1-AP | -0.082 | 0.703 | 22 | Δoverjet | -0.107 | 0.433 | 22 |
| ΔUpperLip-E line | -0.080 | 0.710 | 23 | ΔNose Prominence | -0.103 | 0.450 | 23 |
| ΔGonial Jaw Angle | -0.065 | 0.763 | 24 | ΔPog-NB | 0.097 | 0.475 | 24 |
| ΔOP/SN | -0.063 | 0.769 | 25 | ΔOP/SN | 0.095 | 0.484 | 25 |
| ΔSNB | -0.059 | 0.783 | 26 | ΔSNB | -0.062 | 0.651 | 26 |
| ΔY Axis | -0.046 | 0.830 | 27 | ΔMP/FH | -0.036 | 0.794 | 27 |
| ΔNose Prominence | -0.039 | 0.856 | 28 | ΔWits^#^ | 0.024 | 0.860 | 28 |
| ΔU1/SN | -0.028 | 0.898 | 29 | ΔGonial Jaw Angle | 0.008 | 0.954 | 29 |
| ΔU1/NA | 0.005 | 0.983 | 30 | ΔSNA | 0.003 | 0.982 | 30 |

^#^ ΔANB, and ΔWits were shown as a skewed distribution, the correlations between subjective VAS scores and objective measurements were assessed using Spearman correlation.
